# The Place of Local Field Potentials in Deep Brain Stimulation Programming for Parkinson’s Disease: A Review

**DOI:** 10.3390/brainsci15020116

**Published:** 2025-01-25

**Authors:** Chun Him Shelton Leung, Hugh D. Simpson, Dominic Thyagarajan

**Affiliations:** 1 Department of Neurology, The Alfred Hospital, Melbourne, VIC 3004, Australia; shelton.leung@monash.edu (C.H.S.L.); hugh.simpson@monash.edu (H.D.S.); 2 Department of Neuroscience, School of Translational Medicine, Monash University, Melbourne, VIC 3004, Australia

**Keywords:** Parkinson’s disease, local field potentials, deep brain stimulation, beta oscillations, leads contact selection and programming

## Abstract

Background/Objections: The pharmacological management of Parkinson’s Disease (PD) is often supplemented by deep brain stimulation (DBS) to tackle problems of advanced disease such as motor fluctuation, dyskinesias or medication-resistant tremor. DBS uses high-frequency stimulation with spatially distributed electrodes to produce electrical fields that influence neuronal networks. The programming of such stimulation is complex and time-consuming. Recent technological advancements have enabled DBS systems to record local field potentials (LFPs). In conjunction with biomarker discovery, such as beta oscillations, this shows promise in streamlining the DBS programming process. This review aims to synthesize the current literature investigating LFP characteristics in PD in order to understand the place of LFPs in assisting with DBS programming. Methods: A comprehensive literature search was conducted using databases including OVID MEDLINE, Scopus, and Cochrane Library, resulting in 738 identified articles; 122 studies remained after screening and 87 studies were selected for detailed analysis. Results: Analyzing LFPs clearly has the potential to assist or streamline DBS programming in clinical practice, but there are knowledge gaps and challenges to overcome, especially in the utilization of intraoperative LFPs. Conclusions: More research is required to compare different algorithms that utilize LFPs in DBS programming to identify a simple, practical and time-saving algorithm incorporating reliable LFP biomarkers that will enhance the DBS programming experience for both patients and clinicians.

## 1. Introduction

Parkinson’s Disease (PD) is a progressive neurodegenerative disorder characterized by cardinal motor symptoms including bradykinesia, tremor, rigidity and postural instability. As Parkinson’s Disease advances, symptoms can be challenging to treat with pharmacological therapy alone. Deep brain stimulation (DBS) implantation into the subthalamic nucleus (STN) or Globus Pallidus Internus (GPi) is a highly effective treatment for managing complex motor symptoms in PD. It provides benefits in managing motor fluctuations, troublesome dyskinesia and medications-resistant tremor, and in reducing the overall medication burden [1]. As technology advances, DBS not only provides a therapeutic effect in treating motor symptoms of Parkinsonism, but it also allows for the detection and recording of ambulatory local field potentials (LFPs), which are neurophysiological electrical signals generated by the synchronized activity of neurons in a specific brain region. LFPs have gained significant attention in recent years as potential biomarkers for Parkinson’s Disease, as specific oscillatory frequencies in the beta range, also known as beta oscillations (in the 13–30 Hz range) are found to be abnormally elevated in association with motor symptoms, such as rigidity and bradykinesia [2], and suppressed following pharmacological treatment and DBS [3,4,5]. Analyzing the real-time LFPs gives rise to valuable opportunities for clinicians to personalize stimulation parameters so as to enable more precise lead programming and hopefully enhance the therapeutic efficacy of DBS.

This literature review aims to synthesize current research on the characteristics of LFPs in PD and explore the potential of utilizing LFP biomarkers in DBS programming. The review will cover the following key domains: firstly, the relationship between different LFP biomarkers such as beta oscillations and motor symptoms in Parkinson’s Disease. Secondly, the review will explore the current approach and technology in DBS programming, including the challenges, limitations, and future directions of this technology. Lastly, unifying the current approaches to the application of LFPs in DBS programming, and comparing the current published studies that use different methods and algorithms that integrate LFPs in DBS programming, are also aims. The objective is to first identify the limitations of the current DBS programming technique, then subsequently understand the potentials of incorporating LFP biomarkers in improving DBS programming practice and hopefully improve clinical outcomes for PD patients.

## 2. Methods

The literature search was performed based on the research question “Can local field potentials (LFPs) be used to improve deep brain stimulation (DBS) lead contact selection and programming in Parkinson’s disease patients?” A direct search on the research question was performed in Google Scholar to identify some Gold Set literature. Subsequently, the key search terms included “local field potentials (LFPs)”, “local field activity”, “neural field potentials”, “local potentials”, “beta oscillations”, AND “deep brain stimulation (DBS)”, DBS leads selection”, DBS lead programming”, “DBS electrodes”, AND “Parkinson’s disease”, “Parkinsonism”, “Idiopathic Parkinson’s”, “Parkinson”. Three databases including OVID MEDLINE, Scopus and Cochrane Library were used to ensure a broad capture of relevant literature. Covidence was used to combine the search results, and a total of 738 articles were identified. Abstract screening was performed to filter out irrelevant studies, and a total of 122 articles were found, which captured all of the Gold Set articles. Subsequently, a detailed full-text analysis identified 87 relevant articles included in this literature review.

## 3. Local Field Potentials in Parkinson’s Disease

The potential relevance of LFPs in PD arose in 2003 when Brown et al. reviewed the pattern of neuronal discharges in the basal ganglia of Parkinson’s patients [6]. They described the association between synchronized oscillations at low frequencies (3 to 10 Hz) and Parkinsonian rest and action tremor [6]. Since then, numerous studies have investigated the implications of these synchronization patterns for the clinical state or the severity of symptoms in PD patients. A summary of the different LFP biomarkers has been provided in Table 1. The most consistent finding has been the correlation between STN beta oscillations and contralateral hemibody rigidity and bradykinesia, as measured by parts of the UPDRS part III motor score [7,8,9]. Direct recordings of LFPs in both the STN and GPi using DBS macroelectrodes have shown a synchronization pattern at the beta bands (13 to 30 Hz) in Parkinson’s patients, which was most prominent in untreated the Parkinsonian state [6,7,10,11]. In contrast, the suppression of beta oscillation activities and an increase in lower frequency (theta and alpha bands) activity were demonstrated when patients’ symptoms improved after treatment with either levodopa or DBS [3,5,12]. Hence, some have proposed that a low frequency/beta power ratio can be used as a biomarker for adaptive DBS [13]. Similarly, synchronization at a high gamma band (>60 Hz) was found in PD patients to correlate positively with movement velocity in parallel with fast movements [8], as well as improvement in bradykinesia after treatment with levodopa [9]. Even higher frequency oscillations (>200 Hz) were also found to be prokinetic and to increase at movement onset [12,14,15]. Within the beta frequency, studies found different patterns between low beta (13–20 Hz) and high beta (21–35 Hz) frequencies; low beta was found to be more prominent in the GPi, and was inversely correlated with the severity of bradykinesia and rigidity [2], while it was more sensitive to levodopa suppression [16]. On the other hand, STN high and low beta bands represent different abilities to predict motor symptoms; low beta consistently predicts total Parkinsonian symptoms [17,18,19], while high beta predicts the responsiveness of motor symptoms to PD therapies such as levodopa or DBS [20,21]. It is also important to note that up to 64% of PD patients can demonstrate dual (both low and high beta) peaks within the STN [17].

Regarding other motor features of PD, clinical tremor has been shown to correlate with the tremor frequency band (4–8 Hz), observed shortly after the tremor onset [22], while beta bands were continuously suppressed during clinical tremor maintenance [22]. Recently, Michiel et al. described an integrated pathophysiological pathway for PD tremor known as the dimmer switch hypothesis [23]. This theory integrates the basal ganglia–cortical loop and the cerebello-thalamo-cortical circuit in the generation and maintenance of tremors, which may explain the observation of the tremor frequency in both cortical and STN recordings. The pedunculopontine nucleus (PPN), being the brainstem locomotive center with connections with the basal ganglia and spinal cord, is a structure important for gait control and locomotion. There has been a suggestion that PPN stimulation, aiming to target axial motor symptoms in PD, may improve gait freezing or dysregulation [24]. Subsequently, LFP recordings from the PPN macroelectrodes have shown the attenuation of alpha band activity during gait freezing. In contrast, peaks of alpha power were observed at rest and during unconstrained walking, suggesting a correlation between alpha oscillations and improved gait function [25]. Lastly, peak dose and diphasic dyskinesias have been shown to be associated with theta-alpha activity in the 4–10 Hz frequency range [26,27].

Phase amplitude coupling is a phenomenon that occurs when the phase of slower oscillations frequency couples with the amplitude of faster oscillations, thought to be a mechanism that integrates neural activity in the brain [28]. Beta-gamma coupling between the STN and the primary motor cortex has been found in patients with Parkinson’s disease compared with other movement disorders [29]. This prompts a potential model of a basal ganglia–cortical circuit that could contain sites for stimulation. More recently, Kimoto et al. studied the different beta-gamma PAC patterns measured by scalp electroencephalography in the sensorimotor cortex in Parkinson’s patients with freezing of gait. They found a significant increase in beta-gamma PAC in patients with freezing of gait at the initiation of walking, physiologically distinguishing them from patients without freezing of gait [30]. Although the application of PAC is still unclear in clinical practice, it unlocks the potential for further research to enhance the precision, adaptability and effectiveness of DBS programming.

**Table 1:** Summary of different LFP biomarkers in PD.

LFP Biomarkers	Recording Technique	Recording Location	Associated Motor Symptoms	References
LF oscillations (2–7 Hz)	Macroelectrodes three days post-implantation	STN	Improvement in UPDRS III after levodopa or DBS stimulation	Giannicola et al., 2013 [16]
LF oscillations (4–10 Hz)	Postoperative macroeletrodes	GPi	Improved motor symptoms in the treated state	Silberstein et al., 2003 [31]
Alpha oscillations (7–10 Hz)	Post-operative macroeletrodes	PPN	Improved gait function	Thevathasan et al., 2012 [25]
Theta-alpha activity (4–10 Hz)	Postoperative macroelectrodes	STN	Peak dose and diphasic dyskinesia	Alonso-Frech et al., 2006 [27]
Beta oscillations (8–30 Hz)	Postoperative macroeletrodes	STN	Untreated PD state	Canessa et al., 2016 [11] Kühn et al., 2004 [7]
Beta oscillations (8–30 Hz)	Postoperative macroeletrodes	STN	Contralateral hemibody rigidity and bradykinesia	Neurmann et al., 2017 [32] Kühn et al., 2008 [33] Özkurt et al., 2011 [34]
Low beta bands (13–20 Hz)	Postoperative macroeletrodes	GPi	Rigidity and bradykinesia	Tsiokos et al., 2017 [2]
Low beta bands (13–20 Hz)	Postoperative macroeletrodes	STN	UPDRS III total score	Darcy et al., 2022 [17] Merk et al., 2022 [18]
High beta (21–35 Hz)	Postoperative macroeletrodes before IPG connection	STN	Predicts improvement in motor symptoms with stimulation	Chen et al., 2022 [20]
LF/beta ratio	Macroeletrodes 7 years post-implantation	STN	In a treated state after DBS stimulation	Giannicola et al., 2012 [13]
Gamma (69–90 Hz)	Postoperative macroeletrodes	STN	Correlates with fast movement	Lofredi et al., 2018 [8]
High-frequency band (>200 Hz) PAC	Postoperative macroeletrodes	STN	Prokinetics	Litvak et al., 2012 [12] Lopez-Azcarate et al., 2010 [15]
Tremor frequency (4–8 Hz)	Chronic sensing with macroeletrodes	STN	Rest tremor	Hirschmann et al., 2019 [22]
Beta-Gamma PAC	Scalp EEG	Sensorimotor cortex	Freezing of gait	Kimoto et al., [30]

LF—low frequency; STN—subthalamic nucleus; GPi—Globus Pallidus Internus; PPN—pedunculopontine nucleus; UPDRIS III—Unified Parkinson’s Disease Rating Scale Part III; IPG—implantable pulse generator; PAC—phase-amplitude coupling; EEG electroencephalography.

## 4. Current Approach to Deep Brain Stimulation in Parkinson’s Disease

DBS targets for PD patients primarily include the STN and the GPi, chosen based on the patient’s clinical characteristics. Meta-analysis has shown improvements in motor symptoms of 50.5% for STN-DBS compared to 29.8% for GPi-DBS [35], but very few head-to-head randomized controlled trials directly comparing STN and GPi target outcomes, hence the interpretation of motor function superiority in STN targets is still controversial. Of the two randomized control trials, both studies showed no difference in motor function but a higher risk of adverse effects in STN stimulation, including slower processing speed, verbal fluency and depression [36,37]. Both targets have been shown to improve dyskinesia and motor fluctuations in the long term [38], and STN-DBS provides a further benefit in medication reduction [39], while GPi-DBS appears to entail less risk of causing cognitive impairment or dementia, and is possibly more suitable for older, more frail patients [40]. Stimulation at the ventral intermediate nucleus (VIM) of the thalamus can improve PD tremor, but does not improve other symptoms of PD such as bradykinesia, dyskinesia or rigidity [41].

Initial programming often begins several weeks after DBS surgery, as some believe that clinical benefits may be confounded by the insertional effect (a temporary lesional effect caused by the mechanical positioning of the electrode) and the lower level in impedances due to local edema in the early postoperative period. However, in reality, clinical practices vary, and some clinicians begin programming as early as the day after surgery. Initial programming (usually performed in the medication OFF state) focuses on parameter settings including amplitude, pulse width, and frequency through iterative adjustments and patient feedback, so as to improve efficacy while minimizing adverse effects.

### 4.1. Current Practice for Initial Deep Brain Stimulation Programming

The current standard practice for initial DBS programming is through a “monopolar review” process [42,43], where stimulation is applied sequentially to each contact (or segment) in “monopolar” mode. Monopolar stimulation—also referred to as referential stimulation—refers to a stimulation configuration that utilizes the implanted pulse generator (IPG; also battery, or ‘can’) as an anode, and each individual contact (or segment) of the DBS electrode as a cathode. Stimulation voltage followed by frequency has been shown to be the most important factor in improving Parkinsonian signs [44]. Hence, clinicians usually increase the stimulation amplitude in 0.5 mA/V increments while keeping the pulse width and frequency constant at 60 μs and 130 Hz, respectively. The goal is to identify the lowest amplitude threshold for inducing clinical benefits and the lowest amplitude threshold that induces unwanted side effects, hence identifying the best therapeutic window for each contact [42]. The most useful clinical sign used to assess for stimulation benefit is rigidity, as it usually responds within seconds to STN or GPi stimulation, and is not as confounded as other signs are by other factors such as fatigue, patient’s cooperation, or training [42]. This can be assessed via passive flexion/extension at the wrist joint. Effective stimulation can induce a marked (70%) reduction in muscle tone within 20–30 s [45]. If rigidity is not present (or very minimal), then bradykinesia or rest tremor may be used as alternatives, taking into consideration the possible variable time delay in their clinical response to DBS. Stimulation’s adverse effects can be variable, affecting the somatosensory pathway causing paresthesia, the motor pathway causing muscle contractions, gaze palsy, dyskinesia or dystonia, the autonomic pathway causing dysautonomia, and the limbic system causing depression or mania (or nonspecific effects such as fatigue, malaise or confusion). Somatosensory and nonspecific side effects are usually transient (subsiding several hours after the programming session), while motor side effects usually persist unless the stimulation threshold is reduced. Dyskinesia usually occurs a few hours after programming, and hence it can be difficult to predict during the programming session [42]. Finally, the contact with the largest therapeutic range can be chosen for chronic stimulation, usually beginning from a lower amplitude (1–2 V) with incrementations in subsequent programming sessions. Most DBS systems also allow patients to adjust their own stimulation settings via a patient programmer or handset. In this way, patients can self-titrate the stimulation amplitude up to certain limits set by clinicians. The details of this systemic approach to the initial programming were published in 2016 by Marina and colleagues in a publication known as the Toronto Western Hospital Algorithms [42].

Other considerations made during the initial programming session include checking the impedances for each electrode contact to ensure no hardware problems. Post-operative neuroimaging should be reviewed to exclude surgical complications such as bleeding or infections. Reviewing the locations of the electrode leads is important to ensure no electrode migration or misplacement. In addition, visual software is available that utilizes neuroanatomical information based on fusions of preoperative magnetic resonance imaging (MRI) and postoperative computed tomography (CT) scans. This allows clinicians to precisely visualize the DBS electrodes in a three-dimensional manner in order to facilitate DBS programming [46]. There is evidence that contacts in close proximity to the dorsolateral border of the STN between the upper STN (sensorimotor part) and the subthalamic area containing the zona incerta, fields of Forel, and STN are most effective [47]. In terms of pallidal stimulation, contacts in the dorsal GPi within the globus pallidus pars externa border are most effective [48]. Many clinicians now choose to use this image-based approach in DBS programming, as it has been shown to significantly reduce programming time without compromising motor clinical outcomes or inducing unwanted side effects [46,47,49,50].

### 4.2. Limitations and Challenges in Deep Brain Stimulation Programming

Despite the systematic approach to DBS contact selection and programming, there are significant limitations to be considered. The current limitations primarily revolve around the intricacies of accurately targeting and modulating specific brain regions to achieve maximal clinical benefit. The first programming session can be time-consuming, taking up to one to two hours, followed by frequent subsequent visits for further adjustments, often taking up to 3–6 months (or longer) for the stabilization of symptoms. Furthermore, DBS programming requires a highly trained clinician with extensive expertise in understanding the effects of various DBS stimulation parameters in altering clinical effects, as well as the anatomical variability among patients [51]. The technical nature and complexity of fine-tuning stimulation parameters usually necessitate many years of training in a highly specialized neuromodulation center. This level of expertise is not readily available. This is particularly important when a patient experiences suboptimal clinical benefits or side effects due to the stimulation of non-targeted regions. Different strategies have been developed to guide the management of stimulation-induced dyskinesia, speech impairment, gait impairment and postural instability. This includes reducing the spread of the stimulation or volume of tissue activation (VTA) by using bipolar simulation (when one contact is the cathode and the other anode, causing less current spread) or by using newer directional or segmented electrodes [52]. The other method is interleaving stimulation, in which two preset programs with the same frequency alternate in quick succession to target two distinct clinical symptoms with different therapeutic windows [53,54]. It also benefits from increasing stimulation frequency in the small overlap area, while keeping the frequencies at each core of the stimulation lower. However, this strategy requires more electricity and drains more battery [55]. Low-frequency stimulation (LFS) at <100 Hz may provide benefits for axial symptoms such as speech, gait and balance impairment, but the effect may only be short-term and comes at the cost of worsening appendicular motor symptoms [56]. Shortening the pulse width (below 60 µs) can allow for a significantly wider therapeutic window without compromising UPDRS III motor scores, but may not improve speech symptoms [57]. Stimulation-induced dyskinesia is usually more of a problem in STN DBS, as GPi DBS induces a direct antidyskinetic effect [37]. Some specific programming strategies used to tackle this problem include very slow increments in stimulation amplitude [45], activating the dorsal contacts in the zona incerta above the STN [58], or rescue surgery with GPi DBS if all other methods fail [59,60]. Gait freezing and impairment is a complex, dynamic and multifactorial process affected by a combination of disease progression, the effect of surgery and the stimulation, and other medical comorbidities. In general, STN DBS only improves freezing of gait (FOG) during the OFF phase, and responds well to levodopa [61], highlighting the importance of the levodopa challenge test. On the other hand, GPi DBS may help with ON phase FOG and the preservation of gait function, both of which do not respond to STN DBS [62]. Some other reported strategies besides low-frequency stimulation include the stimulation of the ventral contact of the lead, aiming at substantial nigra pars reticulata (SNr) or interleaving stimulation between the STN and SNr [63]. All these advanced techniques add to an extra level of complexity requiring more specialized expertise and time to troubleshoot various problems. Recent advancements in imaging and more sophisticated DBS technology can be part of the solution to these challenges, and improve patient outcomes.

## 5. Technological Advancement in Deep Brain Stimulation

The three main DBS system manufacturers are Medtronic, Boston Scientific and Abbott. The latest system from Medtronic is the Percept™ PC neurostimulator, released in January 2020 and approved by the FDA in January 2024. The Percept™ RC rechargeable neurostimulator has been FDA- but not TGA-approved. This system features brain signal sensing and recording technology, as well as directional leads, integrating LFP recordings and stimulation within the same DBS device. Medtronic’s DBS system also allows for interleaving stimulation. Boston Scientific’s latest system, named Vercise™ Genus DBS System, was released in 2012 and FDA-approved in 2017. Subsequently, the Vercise Gevia IPG with the Cartesia directional DBS lead was approved in 2019. This system offers multiple independent current control, in which multiple independent current sources generated by the IPG can be delivered to each electrode (different stimulation amplitudes for up to 16 independent outputs at up to two independent frequencies), allowing clinicians to directly control the amount of current delivered to the surrounding tissues, hence adjusting the VTA to a desired shape [64]. Boston Scientific also offers a three-dimensional visual software that allows clinicians to visualize the location of the DBS lead based on preoperative MRI and postoperative CT scans to assist with imaged-based programming. This is known as the GUIDE system. Abbotts Liberta RC™ DBS System was recently approved by the FDA in January 2024, featuring NeuroSphere™ Virtual Clinic, allowing clinicians to remotely program the DBS setting. However, as this technology is not yet widely available, we are yet to determine the practicality and challenges of remote DBS programming. Abbott’s system also offers directional lead and wireless programming via Bluetooth connectivity.

### 5.1. Brain Sensing Technology

Currently, real-time LFP sensing technology (BrainSense) is only offered by the Medtronic Percept™ PC and RC neurostimulators. This technology continuously senses and records brain signals directly from the leads implanted chronically in the ambulatory setting. The technology is designed to record LFPs while simultaneously providing simulation. The recorded signals or data can be stored in the neurostimulator and accessed by clinicians. In theory, this extra piece of information can further inform clinicians and help to tailor DBS treatment to the patient’s needs. During programming, clinicians can perform a survey to search for the beta peak, followed by the real-time streaming of the beta peak while switching on the stimulation; as the stimulation power increases, beta suppression may be observed, which is proposed to correlate with clinical efficacy. Once a therapeutic window is identified, a threshold set-up can be performed to capture out-of-clinic events for up to 60 days, allowing clinicians to review the pattern of beta activity during the next consultation. However, it is important to note that chronic brain sensing can decrease battery lifespan, but the exact amount of energy required for sensing is not known. However, the Percept™ PC device has a projected mean longevity of more than 5 years [65].

### 5.2. Directional DBS

All three manufacturers (Medtronic, Boston Scientific and Abbott) produce directional (or segmented) leads. Traditional cylindrical contacts create approximately spherical electric fields that evenly spread in all directions from the active contact in the monopolar stimulation mode. In contrast, directional leads contain multiple contacts (usually in a circumferential ring form with three individual contacts) around the shaft of the DBS lead, giving clinicians flexibility in selecting an individual contact or a combination of contacts when shaping the direction of the stimulation, known as steering or directional stimulation. This steering technique helps clinicians target the area of interest while avoiding the area that induces undesirable side effects. Directional DBS has been shown to decrease the threshold required for clinical benefit and increase the side-effect thresholds, widening the therapeutic window by an average of 40% [52,66,67]. An international randomized double-blind crossover study conducted by Schnitzler et al., in which patients were switched from traditional omnidirectional stimulation after three months to directional DBS for another three months, showed a mean increase in the therapeutic window by 41%, and an improvement in the UPDRS III motor score by 0.9%. More patients and clinicians preferred the directional period of the study [68].

### 5.3. Closed Loop Systems and Adaptive DBS

The recent advancement in artificial intelligence technology has furthered the progress towards a closed-loop DBS system. Currently, in the open loop system, the stimulation parameters are largely left unchanged in between clinical reviews. Patients have some freedom to adjust the stimulation amplitude (to a certain limit) depending on their symptoms. However, the patient’s disease state and symptoms are of course dynamic. A closed-loop system aims to deliver electrical stimulation according to the changes in biomarkers that correlate with symptoms in real-time, also called adaptive DBS (aDBS). Correlating external symptoms with biomarkers using wristwatches and finger motion sensors in conjunction with aDBS has been shown to be safe and effective, and could reduce side effects such as dyskinesia [69,70]. The commercialization of the Medtronic Percept™ PC now provides a way for clinicians to assess and record LFPs. As described previously, beta oscillations in the basal ganglia are the most widely studied, correlating with bradykinesia and rigidity [2]. Experimental studies that used beta oscillations as a feedback biomarker for the aDBS system showed that they can improve motor control [71] and induce less stimulation side effects, such as dysarthria [72] and dyskinesia [73]. One study used an implanted neural prosthesis on two patients used cortical gamma (60–90 Hz) oscillations as a biomarker. It was designed to decrease stimulation when gamma oscillatory activity was high and increase stimulation when it was low [73]. The result showed a reduction in dyskinesia while maintaining therapeutic efficacy [73]. Subsequently, a meta-analysis and systematic review of 19 studies of aDBS showed a significant improvement in motor function and a reduction in total electrical energy delivered when compared to conventional DBS, but the study identified significant publication bias [74]. While aDBS has the potential to simplify the process of DBS programming and allow more patients to have accessibility to this treatment without the frequent need for specialized neuromodulation expertise, currently, it is faced with issues such as identifying reliable biomarkers that are used to trigger stimulation, in particular axial or non-motor symptoms, technical issues such as the battery life, the precision of sensing electrodes and the computational demands of real-time data processing, and safety issues such as the risk of misinterpretation of signal artefacts or other neural signals. When aDBS systems do become commercialized, clinicians will need to be upskilled to incorporate this technology into their practice. This shifting paradigm is an exciting era in neuromodulation, but more research is needed to address the current issues challenging aDBS.

## 6. Current Algorithms Using LFPs to Guide DBS Contact Selection and Programming

There has been much recent interest in the use of different algorithms that utilize LFPs in DBS programming. The majority of LFP studies focus on STN beta oscillations, as it has been shown that DBS contacts with the maximum beta oscillations recording correlate with the most bradykinesia rigidity improvement [20]. Contact selection comes first in programming. Xu and colleagues found that concordance between the use of a novel biomarker—evoked resonant neural activity—in identifying the optimal contact and expert-clinical programming using monopolar review was up to 80%, but it was only 67% in anatomy-based and 50% in beta oscillations-based lead selection [75], while no clinical outcomes were tested. In another study, the post-operative analysis of LFPs in beta frequency was correlated with optimal lead contact selection [76]. Randomized controlled studies that used the maximal beta peak power for programming have shown at least equivalent clinical outcomes when compared to standard of care [77]. Subsequently, a three-arm randomized crossover trial using beta (in particular low beta)-guided programming vs. image-based programming and traditional monopolar review at three months post-DBS surgery showed significantly reduced time required (by around 40 min) for beta and image-guided programming, with no compromise in clinical efficacy [78]. Longer-term studies have shown similar sustained benefits of LFP-guided directional stimulation at 12 months [79]. A more sophisticated automated algorithm, named the “DETEC” algorithm, which used the weighted average of bipolar recordings with the highest beta band activity to determine the most effective monopolar contacts, showed no difference in clinical efficacy compared to monopolar review, with significantly less time required for programming [80]. Other attempts have been made to use simpler algorithms, for example, by using the linear relationship between the degree of low-beta-band suppression and clinical improvement as a tool to guide DBS lead selection, showing improvement in akinesia and rigidity [81]. Very few studies utilize intraoperative LFPs. The intraoperative measurement of beta LFPs is feasible, although not always recordable [82]. When found, recordings of intraoperative micro- and macroelectrode beta LFPs have been shown to correlate with postoperative beta LFPs, and can even estimate the location of the maximal power of beta oscillations and provide information for the selection of electrode contacts [76,83]. Ozturk et al. conducted a randomized double-blind study comparing the traditional microelectrode single unit activity recordings vs. LFP processing in the localization of STN and the implantation of DBS leads. They found a strong concordance between the two methods in contact localization and clinical improvement (with LFP-based implantation providing slightly greater improvement in motor scores) [84]. Other LFP signals can differ before and after intraoperative stimulation [85]. Dong et al. measured LFP signals using the microelectrode recording needle at different depths, and found differences in gamma oscillations and beta/alpha oscillation ratios before and after stimulation, while beta oscillations did not differ significantly. Subsequently, at 6 months post-surgery, LFP-based programming using either gamma LFPs and beta-alpha oscillation ratios showed no difference in the equivalent daily dose of levodopa, contacts change rate, or quality of life score when compared to the traditional programming method; however, the beta–alpha ratio group showed a significantly larger improvement in UPDRS III score, highlighting beta/alpha ratios as a potential biomarker [85]. Longer-term data that utilize intraoperative LFP recording for post-operative DBS lead selection and programming are still lacking.

## 7. Gaps and Future Needs for an Efficient Algorithm That Utilizes LFPs in DBS

The current literature highlights the great potential to utilize LFPs in DBS programming, with significant knowledge gaps in the utility of intraoperative LFPs. The literature also highlights some significant issues with LFP-based lead contact selection and programming. One major issue is the lack of consensus on optimal biomarkers that can reliably predict outcomes. Most studies have focused on beta oscillations; however, beta oscillations are sometimes not recordable even when clinical symptoms are clearly present, and vary significantly between patients, and over time in individual patients. In addition, some studies have suggested that low beta, beta-to-low frequency or beta-to-alpha ratios may offer improved accuracy [2,13,85], while gamma oscillations may be used to detect dyskinesia [8]. On the other hand, effective biomarkers for tremor or axial symptoms (such as gait impairment) remain elusive. Furthermore, while different algorithms that utilize LFPs have been explored, there has been no direct comparison between them, making it difficult for clinicians to determine the best approach. The complexity of some algorithms also presents a logistical challenge, particularly for clinicians without extensive knowledge of neuromodulation, impeding their adoption in clinical practice. Additionally, most studies that explore the utility of LFPs in DBS programming suffer from small sample sizes of between 10 and 20 patients, limiting the generalizability of their findings. Although monopolar review is currently considered the gold standard in DBS programming, it is time-consuming, subjective, and prone to error, especially when it is based on clinicians’ assessments of rigidity or bradykinesia. It also depends on the patient’s state and tolerability, and can vary over months to years, often necessitating repetition. To move forward, there is a need for a simple, logical, easy-to-understand, and effective algorithm that can simplify the DBS programming process. Such an algorithm should make the process of DBS programming more efficient, and at least as effective (if not more so).

## 8. Conclusions

The current DBS programming method relies heavily on the availability of highly trained clinicians; and even then, the process can be complex, subjective and time-consuming. The synthesis of current studies demonstrates the potential of utilizing LFPs in enhancing DBS lead selection and programming. By integrating real-time LFP analysis, clinicians can tailor DBS parameters quicker and more precisely, potentially improving therapeutic efficacy and reducing adverse effects. However, there are knowledge gaps and practical hurdles that need to be addressed.

At this moment, while the integration of LFPs into DBS programming is a promising advancement, further research and developments are needed to realize its full potential. The identification of robust biomarkers in conjunction with a simple, practical programming algorithm that can be generalized to most PD patients undergoing DBS is a major unsolved problem in the ever-growing field of neuromodulation. Future research should focus on defining these biomarkers, refining these algorithms, and integrating them into future adaptive and closed-loop DBS systems.
